# Schistosomiasis transmission at high altitude crater lakes in Western Uganda

**DOI:** 10.1186/1471-2334-8-110

**Published:** 2008-08-11

**Authors:** Rubaihayo John, Moghusu Ezekiel, Clouds Philbert, Abaasa Andrew

**Affiliations:** 1 Public Health Department, Mountains of the Moon University, P.O. Box 837, Fort-Portal, Uganda; 2 Vector Control Division, Medical Department, Kabarole District, Uganda; 3 Medical Research Council, Entebbe, Uganda

## Abstract

**Background:**

Contrary to previous reports which indicated no transmission of schistosomiasis at altitude >1,400 m above sea level in Uganda, in this study it has been established that schistosomiasis transmission can take place at an altitude range of 1487–1682 m above sea level in western Uganda.

**Methods:**

An epidemiological survey of intestinal schistosomiasis was carried out in school children staying around 13 high altitude crater lakes in Western Uganda. Stool samples were collected and then processed with the Kato-Katz technique using 42 mg templates. Thereafter schistosome eggs were counted under a microscope and eggs per gram (epg) of stool calculated. A semi-structured questionnaire was used to obtain demographic data and information on risk factors.

**Results:**

36.7% of the pupils studied used crater lakes as the main source of domestic water and the crater lakes studied were at altitude ranging from 1487–1682 m above sea level. 84.6% of the crater lakes studied were infective with over 50% of the users infected.

The overall prevalence of *Schistosoma mansoni *infection was 27.8% (103/370) with stool egg load ranging from 24–6048 per gram of stool. 84.3%( 312) had light infections (<100 eggs/gm of stool), 10.8%( 40) had moderate infections (100–400 eggs/gm of stool) and 4.9% (18) had heavy infections (>400 egg/gm of stool). Prevalence was highest in the age group 12–14 years (49.5%) and geometric mean intensity was highest in the age group 9–11 years (238 epg). The prevalence and geometric mean intensity of infection among girls was lower (26%; 290 epg) compared to that of boys (29.6%; 463 epg) (t = 4.383, p < 0.05). Though 61%(225) of the pupils interviewed were aware of the existence of the disease, 78% (290)didn't know the mode of transmission and only 8% (30) of those found infected were aware of their infection status. In a multivariate logistic regression model, altitude and water source (crater lakes) were significantly associated with infection.

**Conclusion and recommendations:**

The altitudinal threshold for *S. mansoni *transmission in Uganda has changed and use of crater water at an altitude higher than 1,400 m above sea level poses a risk of acquiring *S. mansoni *infection in western Uganda. However, further research is required to establish whether the observed altitudinal threshold change is as a result of climate change or other factors. It is also necessary to establish the impact this could have on the epidemiology of schistosomiasis and other vector-borne diseases in Uganda. In addition, sensitisation and mass treatment of the affected community is urgently required.

## Background

Schistosomiasis in Africa is caused by an infection with the blood flukes *Schistosoma mansoni *and *Schistosoma haematobium *whose eggs may be found in faeces or urine respectively. Of the parasitic diseases, schistosomiasis is considered to be second to malaria in global importance with since over 200 million people believed to be infected with the disease and a further 500–600 million people living under constant risk of infection worldwide [1]. In Uganda, *S. mansoni *is of wide public health significance and efforts have been made to map out its distribution within the country [2] for effective control. Like all vector-borne diseases, the distribution of intestinal schistosomiasis depends on the spatial distribution of suitable intermediate host snails. Uganda being on the equator with two rainy seasons every year, the country has many fresh water bodies and climatic conditions that favour the transmission of *S. mansoni *in most areas. However, the epidemiology of schistosomiasis is not only dependent on availability of suitable water bodies but also on the suitability of both climatic and environmental conditions for the schistosomes and the different intermediate host snails. In western Uganda particularly in areas surrounding Lake Albert, the main intermediate host snails for *S. mansoni *are *Biomphalaria stanleyi*, while *Biomphalaria sudanica *have perpetuated transmission in areas around Lake Victoria and Lake Kyoga in Central and North Eastern Uganda respectively[3]. Away from the big fresh water lakes, *Biomphalaria pfeifferi *becomes the dominant intermediate host snail for inland transmission and prefers temporary water bodies [4]. Although transmission of schistosomiasis had been reported in crater lakes elsewhere [5,6] no such data were available on crater lakes in western Uganda. The purpose of the present study was to: (i) establish whether there was schistosomiasis transmission in high altitude crater lakes in western Uganda (ii) describe the epidemiology of infection by age, (iii) describe ecological correlates of infection patterns, (iv)investigate the level of awareness and knowledge of the disease by the community and come up with recommendations for effective control.

## Methods

**Figure 1:**
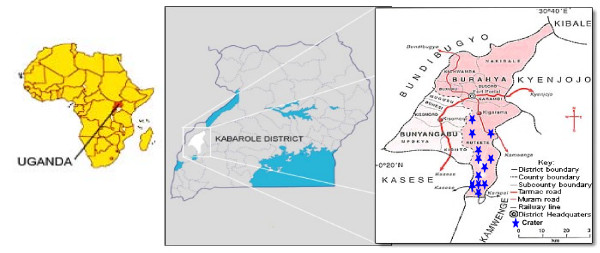
Map showing the location of the crater lakes in western Uganda.

A cross-sectional survey of intestinal schistosomiasis was carried out among primary school pupils aged between 6–18 years living in areas around high altitude crater lakes in Ruteete subcounty, Kabarole District, Western Uganda (Fig 1). Prevalence was determined by analysing stool samples from a cross-section of pupils randomly selected from 6 primary schools representing communities surrounding the 13 crater lakes in the study area. Stool samples were collected from 370 pupils randomly selected from primary one to primary seven classes in the six primary schools and then processed with the Kato-Katz technique using 42 mg templates. Thereafter, schistosome eggs were counted using a microscope and the eggs per gram (epg) of stool calculated. Socio-demographic data and information on risk factors (awareness, source of domestic water, knowledge of transmission and how to avoid the disease) were collected from the same pupils using a semi-structured questionnaire. 13 crater lakes were also sampled for the presence of snail intermediate hosts for *Schistosoma mansoni *which were identified using morphological keys [6]

### Ethical clearance

Ethical clearance to conduct the research was sought from Makerere University medical research and publication ethical committee and Kabarole Local Government Medical department. Prior to the field survey, the study team visited the area to discuss with the local leaders and the schools' administration the objectives of the study. Written informed consent was obtained from the parents and teachers of the pupils before stool samples were collected from them in compliance with the Helsinki Declaration.

### Sample size estimation

Sample size was estimated by using Epi-Info Ver6 StatCalc for population surveys/descriptive studies[7] on the assumption that; a 5% sampling error would be acceptable and 50% schistosomiasis prevalence among a total study population of 4,360 pupils. This gave a sample size of 353 but was increased by 5% to 370 to cater for non-response and missing data.

### Statistical analysis

Data were entered into EPIINFO 6.04(CDC, Atlanta GA), cleaned and analysed in SPSS 10.0 (SPSS Inc., Chicago, IL, USA). Overall and sex-specific prevalence were calculated using descriptive statistics of the sample through frequencies and cross tabulations. To test for differences between geometric means, the student t-test was employed. Strength of association between *S. mansoni *infection and various risk factors was by multivariate logistic regression analysis and calculating the odds ratios (OR) with 95% confidence intervals (CI).

## Results

The prevalence of *Schistosoma mansoni *infection was 27.8% (103/370) with stool egg load ranging from 24–6048 per gram of stool. 84.3%(312) had light infections (<100 eggs/gm of stool), 10.8%(40) had moderate infections (100–400 eggs/gm of stool) and 4.9% (18) had heavy infections (>400 egg/gm of stool). Prevalence was highest in the age group 12–14 years (49.5%) and geometric mean intensity was highest in age group 9–11 years (238 epg) (Table 1). The prevalence of infection was 26% (95%CI: 19.7–33.0%) for girls and 29.6%(95%CI: 23.2–36.7%) for boys. But the mean intensity of infection was higher in boys (463 eggs/gm) compared to girls (290 eggs/gm) (t = 4.383, p < 0.05). It was also found that 36.7% of the pupils studied used crater lakes as the main source of domestic water. 61.5% of the crater lakes studied (Table 2) were infective with over 50% of the users infected. Prevalence was generally higher in children who used crater water at lower altitude than at higher altitude (table 2). All the infective crater lakes were infested with snails which were identified using morphological features as *Biomphalaria pfeifferi*/*sudanica*, due to the current taxonomical problems associated with separation of the two species [6,8].

**Table 1:** Prevalence by age-group

**Age(years)**	**No and % Infected**	**Geometric mean eggs per gm of stool (epg)**	**95% CI**
6–8	8 (7.7%)	107	47–224
9–11	22 (21.4%)	238	138–411
12–14	51(49.5%)	152	105–220
15–17	22(21.4%)	110	78–157

Total	103(27.8%)	152	120–192

**Table 2:** Prevalence of schistosomiasis among school children by source of domestic water

**Crater lake**	**No examined**	**S. mansoni prevalence**	**Altitude (meters)**	**Snails**
Marusi	29	25(86.2%)	1487	*B. pfeifferi*
Kiriruma	4	2(50%)	1536	-
Nyairya	5	3(60%)	1547	*B. pfeifferi*
Lyantonde	18	15(83%)	1551	*B. pfeifferi*
Mwamba	10	5(50%)	1568	-
Rugembe	7	5(71%)	1585	*B. pfeifferi*
Nyabikere	17	2(11%)	1631	-
Mwegenywa	3	3(100%)	1632	*B. pfeifferi*
Kifuruka	6	4(67%)	1636	*B. pfeifferi*
Nyinambuga	9	7(77.8%)	1637	*B. pfeifferi*
Nyamwirima	6	3(50%)	1643	-
Nkuruba	13	0(0%)	1652	-
Nyinabulitwa	12	12(100%)	1682	*B. pfeifferi*
**Others(Springs, wells, etc)**	231	14(6.0%)		

**Total**	**370**	**103(27.8%)**		

Though 61%(224) of the pupils interviewed were aware of the existence of the disease, 78% (289)didn't know the mode of transmission and only 8% (30) of those found infected were aware of their infection status. The results of a multivariate logistic regression model (table 3) showed that altitude and source of water were significantly associated with infection.

**Table 3:** unadjusted multivariate analysis of factors associated with schistosomiasis at high altitude crater lakes in western Uganda

**Variables**	**Sub category**	**No examined**	**No & % infected**	**OR (95%CI)**	**p-value**
Sex	Female	181	44(24.3)	1	
	Male	189	59(31.2)	1.41(0.89–2.23)	0.139
Age	≤ 13	234	60(25.6)	1	
	≥14	136	43(31.6)	1.34(0.84–2.14	0.217
Source of water	Crater	135	88(65.2)	1	
	Spring	105	11(10.5)	0.31(0.91–1.05)	0.061
	Well and others	130	4(3.1)	0.19(0.06–0.613)	0.005
Altitude	1401–1500	29	25(86.2)	1	
	1501–1600	44	29(65.9)	0.31(0.91–1.05)	0.061
	> 1600	66	36(54.5)	0.19(0.06–0.613)	0.005
Awareness of the disease	No	146	42(28.8)	1	
	Yes	224	61(27.2)	0.93(0.58–1.47)	0.747
Knowledge of transmission	No	289	77(26.6)	1	
	Yes	81	26(32.1)	1.3(0.76–2.22)	0.334

## Discussion

The public health significance of schistosomiasis is often underestimated partly because like all helminthic infections, its distribution is usually wide spread with few people having heavy infections and severe disease while the majority are asymptomatic with lighter infections [1,9]. Additionally, the magnitude of the problem is usually underestimated especially in light infections due to the low sensitivity of the Kato-Katz technique which is the commonly used diagnostic technique [9,10]. Schistosomiasis is a neglected tropical disease that can cause death although research shows that rather than mortality, the main outcome of infection is chronic disability [11,12]. The degree of morbidity is usually related both to the intensity of infection and the total duration of the infection. Children of school age are usually the highly vulnerable group and represent the infection status in the population. According to the WHO expert committee report on schistosomiasis, children should be the first target group for intervention because of the detrimental effects the disease has on their growth and development [1]. Early diagnosis and treatment of children shortens the duration of heavy infections thereby reducing the risk of severe disease and childhood disability [11]. In this study 15.7% of the children studied had moderate to heavy infection (>100 egg/gm of stool), most of them had bloody diarrhoea and were at high risk of severe disease. Although awareness on the disease was fairly high (61%), the majority (92%) didn't know their infection status while 78% didn't understand the mode of transmission which probably explains the trend of increased intensity of infection with age. It was also observed that the majority of the community in the study area had no alternative source of safe water as the area was covered by multiple layers of volcanic rocks which made it difficult to provide safe under-ground water. Though there were a few protected springs in the area, they were not accessible to most community members hence the high dependency on crater water for domestic purposes. The children (36.7%) who depend on crater water unknowingly become infected while swimming, fishing and fetching water for domestic use. Although there is a possibility that some children could have acquired the infection by travelling to other areas in Uganda [13], given the level of prevalence and the associated relationship with usage of crater water, its unlikely that all the children who were found infected got the infection from elsewhere. Secondly those other lakes are located far away from these villages where the crater lakes are located (75–150 km away).

Previous studies in Uganda showed that the geographical distribution of *S. mansoni *is restricted to areas at an altitude <1400 m above sea level with annual rainfall above 900 mm [2,8]. However, our findings of 27.8% prevalence of schistosomiasis in crater lakes at an altitude range of 1487–1682 m above sea level disapproves the earlier reported altitudinal threshold limit for *S. mansoni *transmission in Uganda. Though the area studied had an annual rainfall of 1200–1500 mm, it was believed to be a no schistosomiasis transmission area based on altitude[2,14] but according to Appleton [15] it is difficult to establish where transmission is likely to occur based only on altitude without looking at other abiotic factors e. g. temperature, habitat suitability, water velocity and pH. In most cases, temperature-dependent variables seem to be the most important limiting factors defining the transmission threshold rather than altitude per se. Although intestinal schistosomiasis had previously been reported at an altitude of 1900 m above sea level in Lake Bunyonyi south western Uganda but the prevalence was <5%[16]. Elsewhere, intestinal schistosomiasis transmission had previously been reported at higher attitudes. For example, in Ethiopia, the upper altitudinal limit for S. mansoni was reported to be 2000–2225 m above sea level [17,18] while in Kenya the transmission threshold limit was reported at 1800 m above sea level [19]. In this study the risk of infection increased with use of crater water probably because at that height the cater lakes remain the only suitable habitat for the intermediate host snails. However, the risk of infection reduced with increase in altitude possibly because lower temperatures limit parasite development [15].

Therefore, in this era of global warming and climatic change, the epidemiology of temperature-dependent infectious diseases could be changing implying that the altitudinal transmission threshold for *S. mansoni *in Uganda earlier reported [2] may have long changed as shown by results of the current study. There is therefore need for re-defining the current altitudinal transmission threshold for schistosomiasis in Uganda.

However, there were some limitations in this study; first of all it was not possible to establish temporal and spatial variability of abiotic factors (temperature, ph, etc) that would have helped to throw some light on cause and effect of the altitudinal transmission threshold change for *S. mansoni *in this area. Secondly, it was not possible to incriminate the *Biomphalaria *snails that were found in the crater lakes due to lack of facilities for proper handling of infectious snails.

## Conclusion

This study has established for the first time the transmission of *S. mansoni *at an altitude higher than 1,400 m above sea level which was previously considered not possible. It has also established that use of crater water at an altitude higher than 1,400 m poses a risk of acquiring *S. mansoni *infection in western Uganda. However, further research is required to establish whether the observed altitudinal threshold change is as a result of climate change or other factors. It is also necessary to establish the impact this could have on the epidemiology of schistosomiasis and other vector-borne diseases in Uganda. In addition, sensitisation and mass treatment of the affected community is urgently required.

## Competing interests

The authors declare that they have no competing interests.  

## Authors' contributions

RJ: Developed the study design, participated in data collection, analysis and manuscript. CP: Participated in data collection, laboratory work, data entry and manuscript writing. writing. ME: Participated in study design, data collection, laboratory work and manuscript writing. AA: Developed the data analysis plan, was responsible for data analysis and participated in manuscript writing. All authors read and approved the final manuscript.

## Pre-publication history

The pre-publication history for this paper can be accessed here:


